# Geneticization outside genetics’ birthplace: ethical, legal and social implications in the Gulf Cooperation Council region

**DOI:** 10.3389/fgene.2026.1716793

**Published:** 2026-02-18

**Authors:** Safa Shaheen, Mohammed Ghaly

**Affiliations:** 1 College of Health and Life Sciences, Hamad bin Khalifa University, Doha, Qatar; 2 College of Islamic Studies, Hamad Bin Khalifa University, Doha, Qatar

**Keywords:** bioethics, ELSI, GCC, geneticization, genetics, genomics, premarital screening, wholegenome sequencing

## Abstract

Geneticization refers to the growing tendency to explain health, behavior, and identity primarily through genetic terms, often at the expense of social, environmental, and cultural factors. This paper offers a novel contribution to the global discourse on geneticization - a phenomenon extensively examined in Western, predominantly secular contexts, by critically analyzing its ethical, legal, and social implications (ELSI) within the Muslim-majority societies of the Gulf Cooperation Council (GCC) region. As genetic screening programs such as prenatal, newborn, and premarital screening (PMS) gain traction across the GCC countries, often supported by advanced technologies like whole genome sequencing and whole exome sequencing, they encounter not only globally recognized concerns but also distinct religio-ethical and socio-cultural challenges that necessitate engagement with Islamic moral frameworks. Drawing on a close analysis of interdisciplinary literature, the study identifies key ethical tensions surrounding theological sensitivities, informed consent, individual autonomy, genetic discrimination, stigmatization, eugenics, and the management of incidental findings. The paper further highlights legal inconsistencies in PMS mandates and the evolving regulatory landscape governing genomic research across the region. Social implications including high rates of consanguinity, varying levels of public awareness, and community reception of screening programs underscore the need for culturally responsive approaches that align with local values and norms. By foregrounding these region-specific dynamics, this paper advances a more contextually grounded and ethically attuned understanding of geneticization beyond its Western foundations. In doing so, it offers new perspectives for navigating the ELSI of genomics in the GCC, with broader relevance to the Arab world.

## 1 Introduction

The Middle East, bridging Africa, Europe, and South Asia, comprises seventeen countries, with thirteen in the Arab world. This region is recognized as the birthplace of major world religions and plays a historical role as a crossroads of human migration and civilization (MacQueen, 2013). Recent findings emphasize its importance in early human migration out of Africa (Mbarek et al., 2022), yet the genomic structures of the population in this region remain understudied. Within this broader region, the Gulf Cooperation Council (GCC) comprising Bahrain, Kuwait, Oman, Qatar, Saudi Arabia, and the United Arab Emirates, represents a unique socio-political and economic bloc. The 20th-century oil and gas boom stimulated significant economic growth in these countries, enabling the transformation of vast desert landscapes into highly developed urban centers with advanced infrastructure, and propelling some to rank among the wealthiest nations globally (Hvidt, 2013; Khamis et al., 2010). The adoption of Western-inspired technological advancements and luxurious lifestyle, alongside high rates of consanguineous marriage, contributed to a dual burden of lifestyle-related diseases and rare genetic disorders (Klautzer et al., 2014). In response, GCC governments have adopted a range of genetic initiatives such as mandatory screening programs and national genome projects supported by advanced technologies like whole genome and exome sequencing, with aims that include understanding the Arab genome, contributing to global genomic research, and ultimately reducing the incidence of inherited genetic conditions (Saffi and Howard, 2015; Ghaly et al., 2016; Ateia et al., 2023).

Beyond high rates of consanguinity and substantial economic wealth, the GCC region is distinguished by unique socio-cultural and religious characteristics, which collectively exert a significant influence on health practices, genetic research, and the ethical framing of biomedical interventions. Islam is the predominant religion, profoundly influencing social behaviors, attitudes, and ethical decision-making. Arab culture places the family rather than the individual at the core of societal organization, with individuals often living in nuclear and extended family units. Elders are highly respected, and familial hierarchies are typically led by male heads of household (Osman, 2018). As children mature, they assume responsibility for aging parents and inherit familial leadership roles. In this collectivist setting, personal decisions are generally aligned with the common good of the family. In tribal communities, these networks extend further to include influential community leaders (Alahmad et al., 2015). The intersection of rapid technological development, Islamic bioethical frameworks, and unique familial and tribal structures make the GCC a particularly compelling case for studying the ethical, legal and social dimensions of genetic and genomic medicine, and the overall phenomenon of geneticization - a process wherein health and identity are increasingly interpreted through the lens of genetics (Lippman, 1991).

The emergence of geneticization in the Gulf region is shaped by an interplay of financial, socio-political, and religio-ethical factors. Economically, oil-driven wealth has enabled Gulf states to invest heavily in advanced healthcare technologies (Alkhamis et al., 2014) and expand healthcare insurance coverage, integrating genetics into modernization and public welfare agendas. Socio-politically, the widespread practice of consanguineous marriages which are culturally encouraged and neither religiously nor legally prohibited, has been strongly correlated with elevated rates of autosomal recessive genetic disorders; for instance, consanguinity in Qatar exceeds 50%, significantly increasing risk of hereditary conditions (Al-Dewik et al., 2018; Bener et al., 2019). Since there is no alternative socially acceptable route to family formation outside of marriage, premarital genetic screening has emerged as a practical and religiously permissible strategy to reduce the incidence of genetic disorders, thereby alleviating long-term strain on national healthcare systems (Al-Shroby et al., 2021; Bener et al., 2019).

Other commonly implemented screening programs in the region include prenatal, and newborn screening, often supported by advanced technologies such as Whole Exome Sequencing (WES) and Whole Genome Sequencing (WGS) (Tadmouri, 2008). The widespread use of such technologies has contributed to the geneticization process (Lippman, 1991). This reshaping of human experience through DNA analysis raises complex ethical, legal, and social questions, including issues of genetic determinism, discrimination, and theological debates about fate and divine will (ten Have, 2001).

Although the Ethical, Legal, and Social Implications (ELSI) of these technologies have been widely examined since the Human Genome Project (HGP), the majority of this discourse has focused on Western, secular contexts (Thomson et al., 1997). In contrast, there is a noticeable gap in scholarship examining how these implications unfold within Muslim-majority societies, particularly those in the GCC. Although individual ethical and legal issues have been addressed, comprehensive studies integrating all dimensions of ELSI in this context remain limited (El Shanti et al., 2015). Even with previous calls having been made to internationalize and diversify the ELSI research agenda (McEwen et al., 2014; Henderson et al., 2012), these efforts have not led to substantial engagement with ethical deliberations in the Arab world (Ghaly, 2024). This article seeks to address this gap by critically analyzing the ELSI within the GCC by exploring how sociocultural norms, Islamic bioethics, and national legal frameworks, intersect to shape the adoption and perception of genetic technologies in the region.

## 2 Methodology

### 2.1 Study design

This study employed a qualitative interdisciplinary research design, combining systematic document analysis with interpretive normative ethical analysis to examine the ELSI of geneticization in the GCC region. The study is situated within the ELSI research tradition and draws on methods commonly used in bioethics, health law, and science and technology studies to generate context-sensitive ethical insights.

### 2.2 Data sources and selection criteria

Data were drawn from four primary categories of sources, namely peer-reviewed academic literature; national laws and regulatory documents; policy reports and guidelines; and authoritative Islamic bioethical scholarship. Sources were identified through structured searches of academic databases (e.g., PubMed, Google Scholar, in addition to Islamic Studies databases made available by the Qatar National Library), governmental and institutional websites, and citation tracking. Inclusion criteria were: (a) direct relevance to genetics/genomics in the GCC or Muslim-majority contexts, (b) substantive engagement with ethical, legal, or social dimensions, and (c) publication in English or Arabic. Sources focusing exclusively on Western secular contexts without comparative relevance were excluded.

### 2.3 Analytical framework

The analysis proceeded in the following sequential and iterative stages:

- Thematic Coding of ELSI Domains:

All included materials were systematically reviewed and coded using a deductive-inductive approach. Initial deductive codes were derived from established ELSI categories (ethical, legal, social), while inductive coding allowed for the emergence of region-specific themes.

ii. Contextual Ethical Interpretation:

Identified themes were then examined through interpretive ethical analysis, situating ethical tensions within their sociocultural, religious, and legal contexts. Particular attention was paid to points of convergence and divergence between dominant Western bioethical frameworks and Islamic moral reasoning.

iii. Normative Evaluation:

The study applied normative ethical reasoning to evaluate the moral permissibility, justification, and implications of genetic practices in the GCC. This involved assessing ethical claims using Islamic ethical principles alongside widely accepted bioethical norms, without privileging one framework *a priori*. The goal was not to prescribe policy, but to critically examine how ethical reasoning is negotiated in practice within the GCC context and generate an ELSI body of research within this context.

iv. Legal and Comparative Analysis:

Legal texts governing premarital screening and genomic research across GCC countries were analyzed using comparative legal analysis. Differences in scope, enforcement mechanisms, consent requirements, and underlying policy rationales were systematically compared to identify patterns and inconsistencies. This legal analysis was integrated with ethical and social findings to assess how law functions as a mediating force in the process of geneticization.

Several arguments and conceptual frameworks discussed in this manuscript build on the authors’ prior peer-reviewed scholarship in Islamic bioethics and genomics (Ghaly, 2024; Shaheen and Ghaly, 2025), which has systematically examined these issues through sustained empirical and normative engagement. This continuity reflects an evolving research program rather than redundancy, allowing the present analysis to extend, refine, and synthesize earlier insights within a broader ELSI framework.

## 3 Results

### 3.1 Ethical implications

Various forms of genetic and genomic testing have become integrated into individuals’ lives at different junctures. This initiation commences as early as the selection of a life partner, notably through mandatory premarital screening in five of the six GCC countries (all except Oman) (Saffi and Howard, 2015). It extends to the pre-conception phase for couples undergoing *in vitro* fertilization (IVF), during pregnancy through prenatal screening, and immediately after birth with newborn screening. These genetic and genomic procedures have increasingly permeated areas traditionally outside the domain of medicine and clinical genetics, such as the deeply personal process of choosing a marriage partner, highlighting the evident process of geneticization. The ensuing discussion focuses on ethical considerations specific to the GCC context, while also identifying issues with broader global relevance and highlighting the distinct challenges and perspectives that emerge within this regional framework.

#### 3.1.1 Theological sensitivities

Religious beliefs play a crucial role in shaping attitudes toward genetic and genomic screening in the GCC region (Alkuraya and Kilani, 2001; Al Sulaiman et al., 2008). Unlike dominantly secular communities, Muslim societies often emphasize religious practices and the belief in the role of divine will in various aspects of life. In the GCC, religiosity influences decision-making across all domains, including governance, cultural practices, and medical ethics (Harkness and Khaled, 2014). As a result, bioethical principles - particularly those grounded in Islamic values become central when evaluating the ethical implications of genetic and genomic technologies.

The predictive capabilities of genetic and genomic screening are evident across various applications: In PMS they inform spousal selection; in prenatal screening (PNS), they influence decisions regarding the continuation of pregnancy; in preimplantation genetic diagnosis (PGD), they enable the selection of specific traits or the sex of the embryo; and in genome sequencing, they are used to forecast an individual’s susceptibility to future diseases. In Islam, great emphasis is placed on the sanctity of life and the concept of divine predestination (*qadar*), which can shape perspectives on genetic interventions. For some Muslims, the predictive capabilities of these technologies would challenge traditional theological concepts, including fate and thus would consider such genetic interventions as a kind of “playing God.” A few Muslim religious scholars expressed skepticism toward genetic screening, as it may be perceived as an attempt to foretell the future, a capacity attributed only to God. Prior to the advent of such testing, these possibilities were part of the unseen world (*ghayb*), known only to God (Ghaly, 2019). The inherent uncertainty of genomic results further exacerbates these concerns, leading some to question the legitimacy of such interventions within an Islamic framework. For many individuals tested, the outcomes reveal a plausible future, not necessarily a deterministic one, but a potential trajectory for themselves or their offspring. Moreover, theological sensitivities are further complicated by the genetic realities of incomplete penetrance and variable expressivity, whereby the presence of a pathogenic variant does not guarantee disease manifestation or predicts a fixed severity, challenging deterministic interpretations of genetic knowledge and underscoring Islamic emphases on divine will, uncertainty, and moral caution in decision-making.

Addressing these concerns involves highlighting related theological concepts such as the precision in God’s creation, which results in consistent laws that can be detected by human intellect, and divine mercy, emphasizing that knowledge of genetic changes (mutations or variations) allows for intervention and thus would align with God’s will and commandment to minimize suffering. A noteworthy argument is offered by the Kuwaiti religious scholar, Ajil al-Nashmi, who seeks to articulate a theological framework for engaging with modern scientific advancements in emerging fields such as genetic engineering, the HGP, and gene therapy. He reminded Muslims that genetic and genomic advancements could only materialize with God’s will, as nothing in this universe can take place outside the domain of God’s omnipotence (Ghaly, 2019). He contended that Muslims should engage with the advancements of contemporary scientific developments and assess each application by weighing its potential benefits and harms through the framework of Sharia principles (Ghaly, 2019). The lifesaving aspect becomes pivotal in navigating these ethical dilemmas, especially when supported by previous success stories that provide tangible examples of positive outcomes.

#### 3.1.2 Autonomy and informed consent

Autonomy is one of the most central yet contested concepts in bioethics and moral philosophy. Broadly, it refers to the capacity of individuals to make informed and voluntary decisions about their own lives, free from coercion or undue influence. Within Western, dominantly secular context, autonomy is strongly associated with liberal philosophical traditions, particularly Kantian self-legislation and Millian notions of individual liberty, which emphasize rationality, independence, and personal choice (O’neill, 2002; Beauchamp and Childress, 2019).

While this individualistic conception has profoundly shaped biomedical ethics, particularly in the principle of respect for autonomy in informed consent, scholars have highlighted its limitations in multicultural, relational, and religious contexts. For example, the idea of relational autonomy has emerged as an important corrective, emphasizing that autonomy is socially embedded and shaped by networks of care, cultural norms, and interpersonal relationships (Baylis et al., 2008; Mackenzie and Stoljar, 2000).

The concept of informed consent is often interpreted differently in various cultures, with family and community influences sometimes playing a significant role in decision-making (Tucak, 2021). Within the cultural context of the GCC, this concept also presents a significant ethical challenge since it is essential to recognize that informed consent, founded on principles of autonomy and liberalism, may not fully align with the cultural values of the Arab region (Khoury and Akoury-Dirani, 2023).

PMS programs in the GCC region are primarily driven by the goal of reducing the incidence of inherited genetic disorders, particularly the prevalent ones that are correlated to consanguineous marriages, which pose significant burdens on both families and national healthcare systems (Saffi and Howard, 2015; Natarajan and Joseph, 2021; Al Arrayed and Al Hajeri, 2005; Tadmouri et al., 2009). In this context, true informed consent requires individuals to have a clear understanding of the purpose, procedures, and potential consequences of the screening, as well as the freedom to decline without fear of reprisal. However, the mandatory nature of the screening itself can undermine the voluntariness of consent. Familial pressures and social expectations can significantly influence individuals’ decisions, potentially compromising individual autonomy, as was discussed by the Saudi geneticist, Al Aqeel, in a conference paper Al Aqeel (2007), in the context of genetic counseling in the Muslim World. This is in addition to the laws and policies that make the genetic screening procedure sometimes mandatory.

In many Arab countries, the state assumes a protective role, prioritizing collective welfare over individual liberties. This aligns with a utilitarian public health approach, prioritizing the wellbeing of the population. However, this approach inevitably clashes with the principle of individual autonomy. Autonomy is a fundamental principle in medical ethics, affirming an individual’s right to make informed health decisions. However, PMS in the GCC challenges this principle by making genetic testing a prerequisite for marriage, effectively restricting the freedom of choice.

Although PMS programs do not legally prohibit high-risk couples from marrying, the social and familial pressures surrounding the marriage plans *serve as a* significant factor in the decision-making process (Alswaidi et al., 2012). As Pellegrino (1994) argues, true respect for autonomy requires recognizing individuals as moral agents capable of making independent health decisions. When state-imposed PMS limits genuine choice, it raises ethical concerns regarding the extent of state intervention in personal and marital decisions. This approach clashes with ethical principles of self-determination, necessitating a careful balance between public health interests and individual rights in medical ethics and policy formulation.

Furthermore, the rapid advancement of genomic technologies, such as WGS and WES, which are commonly used in national genome programs, raise new ethical challenges related to informed consent. The sheer volume of information generated by these technologies can be overwhelming, making it difficult for individuals to fully comprehend the implications of their genetic data. The challenges of informed consent are further compounded by the complexities of genetic information. The ethical implications of genetic tests expand as the scope of those tests expand. Therefore, informed consent can be an ongoing process, also known as dynamic consent, involving continuous education and support, rather than a single, one-time event.

In this context, it is seen that strong kinship networks and collective decision-making structures shape individuals’ choices, particularly in healthcare and research participation (Nakkash et al., 2017). Ensuring genuine informed consent requires addressing cultural sensitivities, providing culturally appropriate counselling, and empowering individuals to make informed choices (Nakkash et al., 2017). Communicating the nuances of genetic risks, probabilities, and potential outcomes requires specialized expertise and culturally sensitive communication. Ensuring that individuals are fully informed about the purpose, procedures, and potential consequences of screening, and are free to decline without fear of reprisal, is essential to uphold ethical standards. This is particularly important for vulnerable populations, such as women in patriarchal societies, migrant workers, refugees, and patients, who may face coercion or limited autonomy in research participation. For example, Killawi et al. (2014) reported that women of Arabic background frequently engage in discussions with their husbands or family members before providing consent to participate in research studies. This practice reflects the broader socio-cultural framework in which family decision-making plays a central role in healthcare and reproductive choices. Understanding these dynamics is essential for ensuring that informed consent processes are culturally sensitive and ethically sound. Ensuring that these individuals are adequately protected within the consent process is essential to upholding ethical research standards (Nakkash et al., 2017).

According to Nakkash et al. (2017) best practices emphasize a context-specific approach to consent materials and procedures rather than relying on standardized, universal guidelines. Consent documents should acknowledge the diverse and dynamic nature of research populations, ensuring that materials are tailored to participants' linguistic and cultural contexts (Nakkash et al., 2017). Alaei et al. (2013) maintain that if researchers and practitioners demonstrate a deeper understanding of the cultural and contextual factors influencing potential participants and actively respect their beliefs and values, they are more likely to gain trust and cooperation. This cultural sensitivity fosters ethical engagement and enhances the overall effectiveness of the informed consent process.

#### 3.1.3 Genetic discrimination and stigmatization

In the Western context, the potential for discrimination extends beyond social spheres, potentially impacting access to employment, insurance, and other essential services. This makes it crucial to protect individual privacy and implement robust anti-discrimination measures to mitigate these potential harms. In the US, acts like Health Insurance Portability and Accountability Act (HIPAA) and the Genetic Information Nondiscrimination Act (GINA) protect against genetic discrimination in the areas of employment and insurance (Joly et al., 2020).

In the GCC countries, as of now, the genetic information is not linked with the electronic health record that is in the hospitals. In Qatar specifically, every citizen and resident is provided subsidized basic healthcare facilities irrespective of whether they have health insurance (Kaabi et al., 2022). This is seen to be the dominant practice throughout the GCC countries (Khoja et al., 2017). The healthcare records are not accessible to third parties like employers, who usually provide insurance as part of the employment benefits. Neither employers nor insurance companies can ask for genetic testing and they cannot have access to the results of the genetic testing. Even if the genetic data gets linked with the health records, it is very unlikely that employers or insurance companies get access to such data. Thus, one can so far argue that there is no serious fear of genetic discrimination, when it comes to employment or healthcare.

The genetic information collected from volunteers in biobanks is consented to be part of the national genome program. This information is deidentified and stored securely with high end encryption security (Althani, 2015). As precision medicine becomes a reality, linking genetic data with health records would become inevitable, in order to provide optimum healthcare. In such a future scenario, there would arise the need to design laws and policies catering to employment and insurance practices in the region so that genetic privacy is protected. It would be of the utmost importance to secure this data with the highest level of security so that this cannot be illicitly accessed by third parties.

Studies have shown that cultural sensitivities and fears of stigmatization can significantly impact individuals’ willingness to participate in genetic testing (El Shanti et al., 2015; Al Eissa et al., 2024). This is particularly relevant in the GCC, where consanguineous marriages are prevalent, and the disclosure of genetic risks can have profound social consequences (El Shanti et al., 2015; Ghaly et al., 2016). Mandatory PMS programs raise concerns about the potential for stigmatization and discrimination since the identification of individuals or couples as carriers of genetic disorders or infectious diseases can lead to social ostracization, particularly in communities where marriage is highly valued and often considered a social obligation. In contemporary Muslim societies, family or tribal names continue to hold significant social value and are often associated with honor and respect. Consequently, being linked to a hereditary condition can result in serious social repercussions for the entire family or tribe. Due to these potential cultural harms, many individuals do not undergo genetic testing, perceiving the risks to familial reputation as outweighing the potential benefits (Ghaly et al., 2016).

Ghaly et al. (2022) propose a framework for safeguarding the genetic privacy of individuals undergoing PMS by advocating for a need-to-know basis disclosure of test results to prospective partners. To uphold confidentiality, they recommend the implementation of confidentiality agreements in cases where full disclosure of genetic findings is deemed necessary. This approach ensures that sensitive genetic information remains protected, thereby preventing potential misuse, stigmatization, or coercion. Furthermore, by restricting unnecessary disclosure, this strategy aligns with Islamic ethical principles, specifically the prohibitions against slander and backbiting, which emphasize the importance of privacy and discretion in interpersonal and societal interactions (Ghaly et al., 2022).

#### 3.1.4 Eugenics

One of the most controversial aspects of development of the field of genetics has been “eugenics.” Eugenics literally means “good creation” and can be defined as the practice of advocating for the selection of desirable traits to improve future generations (Güvercin and Arda, 2008). Contemporary global ethical frameworks governing biomedical research and genetic interventions have been profoundly shaped by the historical abuses associated with eugenics, particularly those exposed during the Second World War. The Nuremberg trials revealed systematic medical atrocities conducted in the name of racial hygiene and scientific progress, leading to the formulation of the Nuremberg Code, which established voluntary informed consent as a foundational ethical requirement in human research (Shuster, 1997). These principles later informed the Declaration of Helsinki, adopted by the World Medical Association, which continues to serve as a cornerstone of international research ethics (World Medical Association, 2013). In the post-war period, global institutions such as the World Health Organization and UNESCO further developed ethical guidelines explicitly aimed at preventing discrimination, coercion, and the revival of eugenic ideologies in genetics and medicine, emphasizing human dignity, autonomy, and justice (UNESCO, 2005; WHO, 1998). Within this historical context, contemporary concerns about “soft eugenics” in genetic screening programs are not merely theoretical but are rooted in a well-documented legacy of ethical failure, which modern governance frameworks seek explicitly to avoid. Genetic screenings prompt concerns about their potential misuse for soft eugenics, and whether they involve value judgments on the (un)desirability of specific genetic traits (Neel, 1999). To address these concerns, it is crucial to examine the context of these procedures and the traits they target.

In the GCC region, PMS primarily focuses on screening potential couples for hemoglobinopathies, employing a preventive approach to reduce the incidence of these genetic conditions. This proactive strategy raises awareness and informs couples of their carrier status, facilitating informed decisions. The practicality of addressing the future progeny’s health status before pregnancy is emphasized, as decisions become more challenging once an *ex vivo* embryo, through the IVF process, or, even more challenging, once pregnancy has commenced. In the GCC population, whose dominant majority are Muslims, attitudes towards (therapeutic) abortion are influenced by religious beliefs, with increased education on prenatal diagnosis contributing to more openness to pregnancy termination during the permissible period, which is within 120 days after conception, believed to be when the “ensoulment”^1^ process has not yet happened, as agreed upon by the majority of Muslim religious scholars (Alkuraya and Kilani, 2001; Bruwer et al., 2022).

Perceptions towards disability vary globally, influenced by societal norms and socio-economic factors (Hosseinpoor et al., 2013). The decision to bring children with disabilities, including those caused by preventable genetic conditions, into the world is often influenced by parental perspectives. The disability community advocates for awareness and acceptance, aiming to reduce discriminatory practices before or after birth (Al-Hendawi et al., 2022). Urbanization plays a significant role in mitigating the stigma associated with disability in some GCC countries like Saudi Arabia (Shaheen and Ghaly, 2025). However, concerns persist regarding the ethical implications of genetic screening and its potential impact on perceptions of disability. While some view these technologies as a means to minimize suffering, others argue that they may inadvertently reinforce negative biases against individuals with disabilities. A nuanced understanding of the socio-cultural context is therefore essential in evaluating the ethical dimensions of genetic screening and its broader implications for disability rights.

#### 3.1.5 Return of secondary findings and incidental findings

The terms secondary findings and incidental findings (IFs) are interchangeably used as they both are findings unrelated to the primary testing indication. By definition, while secondary findings are purposely analyzed as part of the test, IFs are detected unexpectedly during the analysis (Saelaert et al., 2018). Many ethical questions arise when returning results of a research program-with the first one being -should the results be returned or not, since these are results having serious repercussions in people’s lives. At the individual level, this means disruptions in their day-to-day lives. The burden of having to face the probability of developing a life-threatening disease, especially if it was not expressed phenotypically in any other members of the family, would be one that would be too heavy for many to bear. Familial and societal implications also arise, as mutations may become associated with family or tribal names, affecting social status. Patient autonomy and ethical principles of preventing harm and saving lives are pivotal considerations in this context (Ghaly et al., 2016).

Countries like Saudi Arabia and Kuwait adhere to the American College of Medical Genetics (ACMG) guidelines for disclosing IFs in genomic research (Shaheen and Ghaly, 2025). As part of Qatar Genome Program (QGP), actionable findings for life threatening conditions have begun to be communicated to the research participants such as the *BRCA 1/2* carriers, those who have consented to receive the results (Saleem, 2024; Qatar Genome Program, 2022). There are mutations in genes which cause adult-onset diseases that do not have cure such as mutations in *HTT* gene, *SNCA* gene and the *APOE* gene causing Huntington’s, Parkinson’s and Alzheimer’s diseases respectively (Ciurea et al., 2023). When such mutations are uncovered as part of IFs, there arises an ethical dilemma whether to reveal these findings.

Another important incidental finding which is not related to mutations is the knowledge of the misattributed biological relationship among the family members. This could be in the form of parental consanguinity, misattributed maternity or paternity. The most challenging of these could be the misattributed paternity, also called the MAP event, in which the presumed biological father of the subject is discovered not to be so. In Kuwait, by law it is prohibited to avail paternity testing from private genetic testing labs (Shaheen and Ghaly, 2025). In the other GCC countries where private testing is not available, these results may surface in clinical investigations of rare disorders. The ultimate stance taken in this part of the world is non-disclosure of results (Ghaly et al., 2016).

As far as normative ethical perspectives rooted in the Islamic tradition, Ghaly (2024) proposes a structured framework for addressing IFs in genomic research from an Islamic ethical perspective. Ghaly et al. (2016) also observed that the ethical handling of IFs requires close consultation with research participants or patients and obtaining their consent for the management process. The most recent and comprehensive study by Ghaly (2024) used the fivefold categorization of human acts (*al-Aḥkām al-Khamsah* in Arabic) for the management of IFs from Islamic religio-ethical perspective (Ghaly, 2024). In this system all human acts are classified in terms of their ethical value according to Islamic norms and are classified into five categories: obligatory, recommended, permissible, reprehensible or forbidden (Faruki, 1966). Ghaly (2024) regarded this approach as the most appropriate tool, as it allowed for the classification of human actions based on their moral worth, while being firmly grounded in both metaethics and normative ethics. According to him, this multilayered ethical framework, rooted in Islamic traditions, also intersects with elements of secular moral discourses. Consequently, he posited that the proposed approach is both comprehensive and well-suited for the ethical management of IFs. Given the rapid advancements in recognizing and reporting medically significant IFs, a universal, one-size-fits-all approach is impractical.

Accordingly, Ghaly (2024) emphasizes the necessity of disclosing the possibility of IFs and the disclosure of those with life-threatening implications, such as mutations associated with some types of breast cancer or malignant hyperthermia. From an Islamic viewpoint, the disclosure of such findings is considered obligatory, as it enables individuals to pursue preventive or therapeutic life-saving interventions. Ghaly (2024) also recommends utilizing the minimum gene list developed by the ACMG (Miller et al., 2023) ensuring that medically actionable conditions are identified and managed accordingly. This was also endorsed by El Shanti et al. (2015) in their study on the ELSI in the Arab world with a focus on Qatar. This aligns with certain Islamic scholarly perspectives, which advocate for seeking medical treatment as a means of preserving health and preventing harm (Ghaly, 2024). The disclosure of misattributed lineage is considered reprehensible because the application of modern technologies, such as genealogical DNA testing for distant lineage, often produces inconclusive results and may generate profound socio-political implications, including the potential for social unrest and the destabilization of societal structures (Ghaly, 2024). And as mentioned above, the disclosure of misattributed paternity is prohibited since Muslim scholars agree that safeguarding progeny constitutes one of the higher objectives of Sharia, wherein lineage is not defined solely in biological terms but is legally and ethically grounded in the legitimate marital relationship between the parents (Ghaly et al., 2016).

### 3.2 Legal implications

In the Arab world, regulations pertaining to genetic testing and screening are generally lacking, except for specific procedures such as PMS. Lebanon has a dedicated law regarding genetic testing, Law No.625 of 2004, which explicitly addresses human genetic testing and instructs on taking measures to minimize risk exposure and maximize benefits for the patient (Hamze et al., 2016). UAE Federal Law by Decree No. (49) of 2023, Article 4 stipulates regarding the informed consent aspect of genetic and genomic screening (UAE Government, 2023). Given the existing legal gaps, other countries tend to align with international guidelines, particularly those established by global organizations like the United Nations or the ACMG.

In the GCC countries, legal norms are deeply rooted in the Islamic tradition, with the supreme executive, legislative, and judicial powers typically resting with the central government. The Islamic religio-ethical system (Sharia) serves as the principal source of legislation in these countries (Patrinos et al., 2023). While the UAE distributes power between the federal government and the seven individual emirates, each governed by its ruler, the legal framework remains anchored in Islamic principles (Angell, 2006).

Notably, some GCC countries have enacted laws mandating premarital testing, a facet to be scrutinized in the subsequent section. An overview of the laws pertaining to PMS and different aspects are given in Table 1.

**TABLE 1:** The laws pertaining to PMS in different GCC countries and related aspects.

Countries Aspects	Bahrain	Qatar	Kuwait	Saudi Arabia	UAE
Legislation	Law No. 11 of 2004 - Premarital Screening (Al Arrayed and Al Hajeri, 2005)	Law no. 22 of 2006 - Family Law, Article 18 (Ministry of Justice, 2023)	Family Law No. 31 of 2008 - Section 31 (Ministry of Health, 2008)	Royal Decree No.4/B/54,504 (Al‐Sha’bi, 2023)	Federal Law no.13, Article 10 (Ministry of Health and Prevention, 2020) and Personal Status Law No.28, Article 27 (Ministry of Justice, 2019)
Law Initiation Year	2004	2006; implemented from 2009	2008	2002	2009
Penalties	Fine of 500 BHD (Approx. 1300 USD) for violating screening provisions (Al Arrayed and Al Hajeri, 2005)	No fine specified; No refusal of contract based on medical examination results if parties desire (Ministry of Justice, 2023)	Imprisonment up to 1 year and fine of 1000 KWD (approx. 3200 USD) for violations (Ministry of Health, 2008)	No fine specified; No refusal of contract based on medical examination results if parties desire (Gosadi, 2019)	Sharia courts can refuse marriage contracts in case of at-risk marriages (Kooli and Abadli, 2018)
Name of Premarital Screening program	The Premarital Screening and Counseling Program (Al Arrayed and Al Hajeri, 2009)	Premarital Screening and Genetic Counseling Program (Bener et al., 2019)	The Premarital Screening and Counseling Program (Rouh AlDeen et al., 2021)	Premarital Medical Test (from 2002); Healthy Marriage Program (from 2008) (Gosadi, 2019)	The Premarital Screening and Counseling Program (Saleh and Abd El-Kader, 2022)
Conditions Screened for	Thalassemia, Sickle Cell Disease, G6PD, Rubella; HIV/AIDS, HBV, HCV, Syphilis (Al Arrayed and Al Hajeri, 2009)	Sickle cell anemia, Thalassemia, Homocystinuria, Cystic Fibrosis, Spinal Muscular Atrophy (optional); Hepatitis B (HBV), hepatitis C (HCV), HIV/AIDS, Syphilis and measles (for women) (Al-Dewik et al., 2018)	Thalassemia, Sickle Cell Disease; HIV/AIDS, HBV, HCV, Syphilis (Kuwait Premarital Testing Center, 2024)	Thalassemia, Sickle Cell Disease; HIV/AIDS, HBV, HCV (Gosadi, 2019)	Beta thalassemia, Sickle Cell Anemia, other hemoglobinopathies; HIV/AIDS, HBV, HCV, Syphilis (Abu Dhabi Department of Health, 2015)

#### 3.2.1 A comparative analysis of legal aspects of premarital laws in different GCC countries

Differences in legislation that have been observed in terms of the target population who have been mandated to undergo the PMS–Qatar and UAE require both citizens and residents to undergo the screening whereas Bahrain, Kuwait and Saudi Arabia have mandated this procedure only if either or both of the prospective partners are citizens (Kooli and Abadli, 2018). An interesting point of comparison lies in the core focus that guided the formulation of these laws. In the cases of Qatar and Kuwait, PMS has been made compulsory under the family law, signifying a central emphasis on family (Ministry of Justice, 2023). Conversely, Saudi Arabia initiated the program as a premarital medical examination, later renaming it as the “Healthy Marriage Program”, thereby shifting the focus from a medical examination to the concept of ‘marriage’ (Ministry Of Health Saudi Arabia, 2024). In the UAE, the legal framework revolves around public health, encompassing family health as a component. Additionally, there is a separate personal status law addressing matters pertaining to marriage and family relations (Ministry of Justice, 2019). Oman’s approach is particularly interesting, where the PMS procedure isn’t mandated, but it is included as an optional provision within the “Child Law.” Notably, the focus here is on the best interests of the child, as opposed to emphasizing marriage, family, or public health, as observed in the other countries (Al Kamyani and Al Hinai, 2018).

The various PMS and counselling programs that have emerged as an extension of these laws are mentioned in Table 1. Across the different GCC countries, it has been observed that the procedure of PMS is relatively the same: once a couple decides to get married, they must consult the clinic authorized to do this procedure and make an appointment. The basic parameters such as height, weight etc. are measured, family history is assessed, blood samples are drawn, and screening is done for conditions specific to the certain country, which are usually the hereditary disorders prevalent in the country and certain infectious diseases. In specific, the following tests are usually conducted: Complete Blood Count (CBC), sickle cell test, hemoglobin electrophoresis, and screening for infectious diseases.

Couples are provided with their results and also counselled separately by physicians/geneticists in the health center. They are given a sealed envelope that states that the couple has undergone premarital counseling, which has to be handed to the religious authority officiating the marriage. The report is only to show that the couple has undergone the mandatory PMS and counselling; this does not have any genetic status of the couples if they are not affected. If they are affected, the report mentions the genetic compatibility status, without giving details about the exact genetic risks to protect the couple’s genetic privacy. The medical professionals who come across the results are bound by confidentiality principles not to reveal the results to anyone except the patients themselves. Kuwait Family law has ensured the protection of privacy and confidentiality of patients by penalizing the personnel involved in confidentiality breach (Ayed, 2008). The various articles coming under the premarital laws of other GCC countries have not covered this aspect.

Some of the nuances that stand out in the various mandatory PMS programs are seen in terms of whether they are allowed to proceed with the marriage, and if so, the conditions under which they are allowed to proceed. As mentioned above, in Kuwait, the age of the female undergoing the test is taken into consideration (Kuwait Premarital Testing Center, 2024), whereas in the UAE, the final decision rests with the judicial department, unlike in other countries where this screening procedure has been mandated (Emirates Health Services, 2021). If the couple is not allowed to proceed with marriage based on their genetic incompatibility, this would eventually interfere with the ethical principle of autonomy.

#### 3.2.2 Laws regarding implementation of genomic research

Codified laws regarding all aspects of other types of genetic testing are seen to be lacking in this region except for UAE. Federal Decree Law No. 49 of 2023 concerning the Regulation of Human Genome Use provides a comprehensive legislative framework in the UAE to govern the ethical, secure, and responsible utilization of genetic data (UAE Government, 2023). The law is designed to safeguard individual privacy, prevent genetic discrimination, and prohibit unethical or commercial exploitation of genome-related information. The Department of Health in Abu Dhabi has released the policy on genomics, which outlines the strategic directions for the use of genomic data and technologies to improve health outcomes in an ethical way. This policy is directed at all the stakeholders involved with genomics research and development (Abu Dhabi Department of Health, 2022). With respect to issues related to human genetic and genomic research, The Ministry of Public Health of Qatar has issued policy guidelines for “The Design, Ethical review and conduct of genomic Research in Qatar”. The purpose of these guidelines is to provide practical assistance to researchers in the design and conduct of their projects as well as to Institutional Review Boards in the review and oversight of the projects related to genomics (Ministry of Public Health, 2018). Although many governmental bodies and research institutions across the GCC have stipulated guidelines regarding genetic and genomic research, much work has still to be done in this area. There is also an ambiguity regarding the specification of age of consent regarding voluntary genetic testing.

With regards to genetic discrimination, as of now, the Middle East is a region with minimal policy making activities as was observed by Joly et al. (2020). Distinct approaches have been adopted by different countries with respect to protecting against genetic discrimination-varying from broad human rights-based statutory prohibition to reliance on existing traditional legal practices such as privacy law, current bases for nondiscrimination, insurance law, and disability law. While drafting policies for the GCC region, a main factor would be the influence of Islam as opposed to the other regions of the world where policies are prevalent. As of now, the GCC countries do not have a model which they can fully adopt in this regard. Certainly, the basic framework comprising of broad principles of human rights, insurance and disability laws can be adopted, and adapted according to the needs of the region.

### 3.3 Social implications

When studying the social implications of genetic and genomic technologies, it is essential to involve the various stakeholders, including the general public, and study their awareness, knowledge, acceptance, expectations, and attitudes toward the technology in question (Jensen et al., 2019). Many studies have been done in the GCC region to assess these factors that play a major role in analyzing the impact/implications of these technologies in question which will be mentioned below.

#### 3.3.1 Consanguinity

Consanguineous marriage is defined as a union between individuals who are second cousins or closer and is a culturally embedded and socially accepted practice in many parts of the GCC region. Rates of consanguinity remain among the highest in the world, with prevalence ranging from 25% to over 60% in some GCC countries (Tadmouri et al., 2009; Hamamy, 2012). In the GCC region, where large family networks and tribal affiliations are influential, consanguinity is not only common but often preferred due to its socio-cultural and economic benefits. However, these practices pose significant challenges for public health, necessitating proactive and culturally sensitive genetic screening strategies.

While consanguineous marriages increase the risk of recessive genetic disorders, they are often deeply rooted in cultural and familial traditions (Hamamy, 2012). Hamamy (2012) asserts that the promotion of genetic screening programs must be approached with sensitivity, recognizing the social and cultural significance of these practices. Simply discouraging consanguinity without offering culturally appropriate alternatives may lead to resistance and mistrust. Instead, programs should focus on providing accurate information, genetic counseling, and support to families, empowering them to make informed decisions within their cultural context. The influence of familial expectations and potential stigmatization of carriers can complicate decision-making, especially since consanguinity is deeply rooted in social norms (Raz, 2009).

Genetic counseling is seen to serve as a critical interface between medical knowledge and social norms. In consanguineous contexts, counselors must navigate family dynamics, religious beliefs, and limited genetic literacy to facilitate informed and non-coercive decision-making. Ensuring culturally competent counseling that incorporates local values is essential to the ethical success of screening programs (El Shanti et al., 2015).

Public education campaigns are also vital in increasing awareness about the genetic risks of consanguinity and the benefits of early screening. This can lead to more voluntary participation and reduce the stigma associated with being a carrier. Involving religious leaders and community figures in educational efforts regarding consanguinity could prove to be effective in enhancing trust and acceptability.

#### 3.3.2 Knowledge, awareness and acceptance

Public awareness of genetic and genomic screening procedures in the GCC region often hinges on mandatory procedures like PMS, creating routine but vague familiarity. Misconceptions arise from media influence, sometimes leading to extreme views regarding genetics and genomics. In highly consanguineous populations, education becomes crucial on consanguinity effects, abortion risks, rare diseases, and quality of life.

Studies show varying knowledge levels among university students. In Qatar, even after a decade of PMS initiation, knowledge remained relatively low, according to a study conducted among 476 Qatar University students, with 41.4% supporting genetic testing for public health (Al-Shafai et al., 2022). Kuwait University students, despite a positive PMS attitude, struggle to comprehend its necessity (Al-Enezi and Mitra, 2017). Oman’s high school students favor PMS, but awareness about specific diseases tested is lacking, according to a cross-sectional study conducted in 10 public high schools in Muscat (Al-Kindi et al., 2019).

Saudi Arabia, a key focus, reveals mixed knowledge levels. Female students at King Saud University exhibited fair knowledge (Khalil et al., 2014), while others lacked specifics such as the diseases being tested for (Al-Aama et al., 2008). Studies highlight the population’s high awareness but insufficient knowledge, contributing to consanguineous marriages. Educational programs prove effective, improving understanding and attitude regarding these initiatives. In the UAE, Ajman University students showed acceptable knowledge regarding thalassemia screening (Elsadek et al., 2022), with initiatives by institutions and associations promoting awareness.

Overall, mandatory procedures garner acceptance, but knowledge gaps persist, especially in understanding specific genetic aspects. Educational interventions and targeted initiatives, as seen in Saudi Arabia (Ibrahim et al., 2011) and the UAE (Laurance et al., 2014), are effective in improving awareness. Proper documentation of disease prevalence and consanguinity rates is essential for accurate assessments. In the absence of certified genetic counselors, medical personnel step in, emphasizing the need for comprehensive training in genetic counseling services.

#### 3.3.3 Impact, outcome and effectiveness

Assessing the societal impact, outcome and effectiveness of genetic screening programs in the GCC region are crucial for refining existing initiatives. Nationwide screening programs face the challenges of feasibility, accessibility, and cost, but the GCC countries have generally addressed these concerns.

Surveys conducted by the QGP reveal a strong public willingness to contribute to the national genome program (Abdul Rahim et al., 2020). In the outcome studies, usually prevalence of the different types of hemoglobinopathies is estimated from the population that underwent the testing (Memish and Saeedi, 2011; Gosadi, 2019; Bahram et al., 2023). One drawback of these estimates is that only disease prevalence in the population of marriageable age is estimated. This cannot be stated as the prevalence rate in an entire population or country unless nationwide screening programs are conducted to examine such numbers.

The effectiveness of PMS programs is key, aiming to reduce at-risk marriages and, consequently, the prevalence of hemoglobinopathies. Bahrain’s PMS program was deemed as successful since, in the 1980s, 2% of the Bahraini population was affected by Sickle Cell Disease (SCD) and this was reduced by half, a decade later due to education and premarital testing programs introduced by the Government (Al Arrayed and Haites, 1995). But a study conducted by Almutawa and Alqamish (2009) showed that more success should be aimed for since 17 out of 30 high-risk couples proceeded with marriage despite genetic counseling between April and May 2006. This sentiment was reiterated by a recent retrospective study conducted on at-risk couples referred for premarital counselling during the time period of 2018–2020 which showed that out of 159 at-risk couples, 107 couples (67%) proceeded with marriage despite counselling (Bahram et al., 2023). 8 out of 20 infants born to couples after spontaneous conception were affected with hemoglobinopathy (Bahram et al., 2023).

In Kuwait, the PMS program achieved success, convincing over 50% of at-risk couples not to proceed with marriage (Rouh AlDeen et al., 2021). A factor that contributed to the success was the offering of free Preimplantation Genetic Testing (PGT) for couples with incompatible results, preventing the birth of affected children (Rouh AlDeen et al., 2021). Saudi Arabia’s PMS program exhibited varied outcomes. Initial data indicated limited success in reducing at-risk marriages (Alswaidi et al., 2012), but subsequent years saw a marked decrease in such marriages (Memish and Saeedi, 2011).

Factors influencing the effectiveness of PMS in Saudi Arabia include the timing of the test, lack of disease impact awareness, belief in fate, inadequate genetic counseling services, and preexisting emotional bonds (Alswaidi et al., 2012; Algiraigri, 2021). Education on alternatives like IVF and PGT is essential so that couples moving ahead with at-risk marriages can have informed reproductive choices. In the UAE, studies demonstrated a reduction in annual births with thalassemia, indicating the effectiveness of the PMS program (Belhoul et al., 2013).

## 4 Discussion

This study delved into the ELSI of geneticization within the GCC region, a context marked by a distinctive interplay of rapid technological advancement, Islamic bioethical principles, and specific socio-cultural norms, such as high rates of consanguinity. The analysis revealed that the integration of genetic and genomic technologies into the lives of individuals in the GCC countries presents a multifaceted landscape.

The ethical dimensions of geneticization in the GCC are particularly complex, necessitating a delicate balance between upholding individual autonomy and respecting cultural sensitivities. PMS programs, while aiming to reduce the prevalence of genetic disorders, raise concerns about informed consent, given the influence of familial pressures and social expectations in the region. The uncertainty of genetic results and the complexities surrounding the return of secondary or incidental findings further complicate ethical decision-making (El Shanti et al., 2015). Moreover, the potential for genetic discrimination and stigmatization necessitates robust safeguards to protect individual privacy and confidentiality.

The study also highlighted the significant role of religious beliefs in shaping attitudes toward genetic and genomic screening in the GCC. Islamic bioethical principles, emphasizing the sanctity of life and divine predestination, influence the acceptance and interpretation of genetic technologies. Concerns about eugenics and the potential misuse of genetic screenings to target specific traits require careful consideration within the GCC context, where PMS for hemoglobinopathies is a common practice.

The legal landscape in the GCC reflects the influence of Islamic tradition, with varying approaches to regulating genetic testing and screening. While some countries have enacted specific laws, particularly concerning PMS, others rely on broader legal principles or international guidelines. Variations in legislation and implementation highlight the need for context-specific legal frameworks that address the unique challenges posed by geneticization in the region (El Shanti et al., 2015).

Socially, the GCC region grapples with issues such as consanguinity, public awareness and acceptance of genetic technologies, and the impact and effectiveness of screening programs (Al-Gazali et al., 2005). While mandatory screening programs have gained acceptance, knowledge gaps persist, and culturally sensitive approaches are needed to address consanguinity and enhance public understanding. Assessing the effectiveness of screening programs and addressing challenges related to feasibility, accessibility, and cost is crucial for optimizing their impact.

This study has certain limitations that should be acknowledged. Firstly, this paper is based on an analytical study of existing literature, which may introduce inherent biases present in the selected sources. The scope of the study is limited to the GCC region, and findings may not be generalizable to other populations or cultural contexts. Furthermore, the study focuses on the ELSI of geneticization, and other factors that may influence the adoption and perception of genetic technologies in the GCC, such as economic or political influences, were not explored in depth. Also, the diversity within the GCC countries in terms of cultural, legal, and healthcare system differences was not fully explored. While the study highlights regional commonalities, it does not systematically differentiate between individual GCC states, which may mask intra-regional variations in policy implementation or public attitudes. This study offers a preliminary overview of the ELSI associated with geneticization in the GCC region. It does not purport to provide an exhaustive account, given the complexity and breadth of the topic, which requires more extensive and in-depth investigation in future research.

## 5 Conclusion

The findings of this study underscore the importance of context-specific approaches to geneticization in the GCC region. Balancing individual autonomy with public health interests, addressing ethical dilemmas related to uncertainty of IFs, and navigating the complex interplay of religious, cultural, and legal factors require ongoing dialogue, ethical reflection, and adaptation of policies. By acknowledging the unique socio-cultural and religious landscape of the GCC, this analysis contributes to a more nuanced understanding of the challenges and opportunities presented by genetic and genomic technologies in the region.

A nuanced approach is required, one that balances scientific progress with respect for traditional beliefs and values. This involves engaging with religious leaders and scholars, providing culturally appropriate genetic counseling, protecting individual privacy and confidentiality, and ensuring that genetic screening programs are used ethically and responsibly. By fostering open dialogue and promoting a deeper understanding of the intersection between genetics, culture, and religion, the GCC region can navigate the challenges and opportunities presented by these transformative technologies. In navigating the complex landscape of genetic and genomic technologies, the GCC countries are poised to contribute to global discussions on ethical practices, legal frameworks, and societal acceptance. As these technologies advance, ongoing dialogue and adaptation of policies will be essential to ensure that the benefits are maximized while respecting individual rights, religious and cultural values.

## Data Availability

The original contributions presented in the study are included in the article/supplementary material, further inquiries can be directed to the corresponding author.

## Footnotes

1 In Islamic theology and jurisprudence, *ensoulment* refers to the moment at which the soul is believed to be breathed into the developing fetus, marking a significant transition in its moral and legal status. This concept is primarily derived from prophetic traditions describing embryonic development in stages of 40 days each, after which an angel is sent to breathe the soul into the fetus and record its destiny. While some jurists have interpreted these traditions to place ensoulment at 40 days, the dominant opinion among classical and contemporary Sunni scholars locates ensoulment at approximately 120 days after conception, after which the fetus is generally accorded full moral sanctity.
